# Phosphorylation of Lamin A/C at serine 22 modulates Na_v_1.5 function

**DOI:** 10.14814/phy2.15121

**Published:** 2021-11-21

**Authors:** Michael A. Olaopa, Tomohiko Ai, Bo Chao, Xiangshu Xiao, Matteo Vatta, Beth A. Habecker

**Affiliations:** ^1^ Department of Chemical Physiology and Biochemistry Oregon Health & Science University Portland Oregon USA; ^2^ Krannert Institute of Cardiology Department of Medicine Indiana University School of Medicine Indianapolis Indiana USA; ^3^ Department of Clinical Laboratory Medicine Juntendo University Tokyo Japan

**Keywords:** cardiac conduction disease, Lamin phosphorylation, Na_v_1.5

## Abstract

Variants in the *LMNA* gene, which encodes for Lamin A/C, are associated with cardiac conduction disease (CCD). We previously reported that Lamin A/C variants p.R545H and p.A287Lfs*193, which were identified in CCD patients, decreased peak *I*_Na_ in HEK‐293 cells expressing Na_v_1.5. Decreased peak *I*_Na_ in the cardiac conduction system could account for patients’ atrioventricular block. We found that serine 22 (Ser 22) phosphorylation of Lamin A/C was decreased in the p.R545H variant and hypothesized that lamin phosphorylation modulated Na_v_1.5 activity. To test this hypothesis, we assessed Na_v_1.5 function in HEK‐293 cells co‐transfected with *LMNA* variants or treated with the small molecule LBL1 (lamin‐binding ligand 1). LBL1 decreased Ser 22 phosphorylation by 65% but did not affect Na_v_1.5 function. To test the complete loss of phosphorylation, we generated a version of *LMNA* with serine 22 converted to alanine 22 (*S22A‐LMNA*); and a version of mutant *R545H‐LMNA* that mimics phosphorylation via serine 22 to aspartic acid 22 substitution (*S22D‐R545H‐LMNA*). We found that *S22A‐LMNA* inhibited Lamin‐mediated activation of peak *I*_Na_ by 63% and shifted voltage‐dependency of steady‐state inactivation of Na_v_1.5. Conversely, *S22D‐R545H‐LMNA* abolished the effects of mutant *R545H‐LMNA* on voltage‐dependency but not peak *I*_Na_. We conclude that Lamin A/C Ser 22 phosphorylation can modulate Na_v_1.5 function and contributes to the mechanism by which *R545H‐LMNA* alters Na_v_1.5 function. The differential impact of complete versus partial loss of Ser 22 phosphorylation suggests a threshold of phosphorylation that is required for full Na_v_1.5 modulation. This is the first study to link Lamin A/C phosphorylation to Na_v_1.5 function.

## Graphical abstract

Lamin A/C Ser 22 phosphorylation can modulate Nav1.5 function and contributes to the mechanism by which R545H‐LMNA alters Nav1.5 function in cardiac conduction disease. Abolishing phosphorylation with S22A‐LMNA reduces channel function, but decreasing phosphorylation with the small molecule LBL1 does not, suggesting a threshold of Ser 22 phosphorylation required for Nav1.5 modulation. Mimicking phosphorylation with S22D‐R545H‐LMNA abolished the effects of mutant R545H‐LMNA on voltage‐dependency but not peak *I*_Na_.

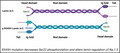

## 1 INTRODUCTION

Laminopathies are a broad spectrum of diseases involving cardiac and skeletal anomalies attributed to mutations in the *LMNA* gene, which encodes for Lamin A/C. These disorders comprise over 12 clinically heterogeneous syndromes (Bertrand et al., 2011). Lamin A/C is a type‐V intermediate filament that forms the nuclear lamina underlying the inner membrane of the nucleus (Houben et al., 2013). *LMNA* mutations are highly prevalent in patients with cardiac conduction disease (CCD), often associated with eventual dilated cardiomyopathy (DCM; Anselme et al., 2013; Barra et al., 2012; Hermida‐Prieto et al., 2004; Keller et al., 2012; Malek et al., 2011; Mounkes et al., 2005; Olaopa et al., 2018; Saj et al., 2010; Shaw et al., 2002; Zaragoza et al., 2016). A clinical study of familial autosomal DCM found that approximately 33% of patients were positive for *LMNA* mutations (Arbustini et al., 2002). *LMNA* patients require implantable cardioverter‐defibrillator or pacemaker therapy to prevent cardiac arrest and sudden death (Anselme et al., 2013; Kumar et al., 2016; Olaopa et al., 2018). The clinical presentations of these patients, including atrioventricular (AV) block and progressive CCD, are similar to those observed in disorders caused by mutations in the *SCN5A* gene (Olaopa et al., 2018; Wang et al., 2002), which encodes for the cardiac sodium channel (Na_v_1.5).

Na_v_1.5 is primarily localized within the sarcolemma, in contrast to the nuclear localization of Lamin A/C. Mutations in *SCN5A* are a major cause of AV block, sick sinus syndrome, progressive CCD, and eventual DCM leading to sudden death (Amin et al., 2010; Chockalingam et al., 2012; Holst et al., 2010; Lee et al., 2016; Makita, 2009; Samani, Ai, et al., 2009; Samani, Wu, et al., 2009; Shuraih et al., 2007). Due to the similarities between clinical presentations of disorders associated with both *LMNA* and *SCN5A* mutations, studies by our group and others have sought to functionally link variants in Lamin A/C to Na_v_1.5 activity (Liu et al., 2016; Markandeya et al., 2016; Olaopa et al., 2018). Our group reported that two Lamin A/C variants (p.R545H and p.A287Lfs*193), found in patients with CCD, significantly decrease peak sodium current (*I*_Na_) and shift the voltage‐dependency of steady‐state inactivation of Na_v_1.5 (Olaopa et al., 2018). The p.R545H variant is due to a missense point mutation (c.1634G>A); while the p.A287Lfs*193 variant is caused by a single nucleotide deletion (c.859delG) and subsequent frame shift, leading to a premature termination codon (Olaopa et al., 2018).

Although the transcriptional and post‐translational regulation of Lamin A/C within the heart is not fully understood, its phosphorylation has functional importance (Buxboim et al., 2014; Haas & Jost, 1993; Kochin et al., 2014; Mitsuhashi et al., 2010; Swift & Discher, 2014; Torvaldson et al., 2015; Wu et al., 2011). Phosphorylation at serine 22 (Ser 22) plays an important role in cell cycle regulation, nuclear stability, and signaling between the nucleoskeleton and cytoskeletal structures––the LINC (Linker of Nucleoskeleton and Cytoskeleton) complex (Buxboim et al., 2014; Kochin et al., 2014; Osmanagic‐Myers et al., 2015; Swift & Discher, 2014; Torvaldson et al., 2015). Additionally, a p.S22L Lamin A/C variant was identified in a genetic screen of cardiac transplant patients with DCM (Pethig et al., 2005), indicating a clinically relevant role for Ser 22 in cardiac disease. Thus, we sought to determine if Ser 22 is involved in the mechanism by which the p.R545H variant affects Na_v_1.5 function, with the aim of identifying a potential therapeutic target for CCD patients with similar *LMNA* mutations. We used a novel small molecule (lamin‐binding ligand, LBL1) that binds Lamin A/C in the N‐terminal region encompassing Ser 22 (Chao et al., 2017; Li et al., 2018), as well as genetic (Ser 22 modification) approaches to modulate Ser 22 phosphorylation and determine its role in Na_v_1.5 function.

## 2 MATERIALS AND METHODS

### 2.1 Cloning and generation of plasmids

**FIGURE 1:**
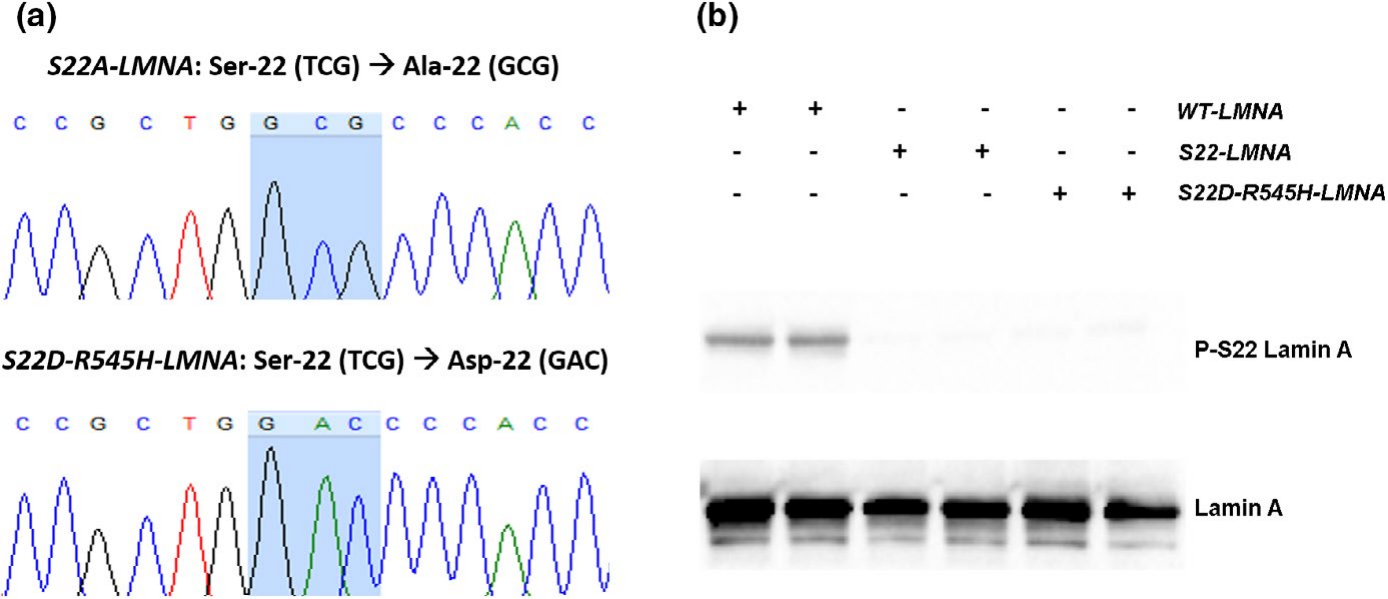
Confirmation of *LMNA* phosphorylation plasmids. (a) Electropherographs of sequencing results confirming mutagenesis of targeted codon (blue highlight) for each respective *LMNA* phosphorylation plasmid. (b) Western blot on protein lysates extracted from HEK‐293 cells transfected with each respective *LMNA* plasmid

The cDNA of wild‐type *LMNA* (ORF NM_005572) was purchased (OriGene) in a pCMV6‐AC‐GFP (C‐terminus fused) vector backbone. Mutant *R545H‐LMNA* and phosphorylation *LMNA* constructs (*S22A‐LMNA* and *S22D‐R545H‐LMNA*) were generated using site‐directed mutagenesis kit (Qiagen). We substituted alanine (GCG) for serine (TCG) at position 22 (p.S22A) in wild‐type *LMNA* to prevent phosphorylation; to mimic phosphorylation in the *R545H‐LMNA* mutant we substituted aspartic acid (GAC) for serine (TCG) at position 22 (p.S22D) in the *R545H‐LMNA* plasmid. DNA sequencing and western blot analyses confirmed successful mutagenesis (Figure 1).

### 2.2 Cell culture and transfection

HEK‐293 cells were grown and maintained in DMEM media (Thermo Fisher), supplemented with 10% fetal bovine serum (ATCC). Cells were serum‐starved overnight and transiently transfected with identical amounts of plasmid DNA using Effectene (Qiagen). Cells were treated 24 hrs post‐transfection with vehicle dimethyl sulfoxide (1% DMSO), 5 or 10 µM LBL1. Cells were harvested 24 hrs post‐treatment or 48 hrs post‐transfection.

### 2.3 Protein isolation and western blot

Protein was isolated in lysis buffer (in mM): Tris 20, etheylenediaminetetraacetic acid (EDTA) 10, NaCl 100, 1% SDS, 1× complete protease inhibitor (Roche), and 1× phosphatase cocktail inhibitor (Sigma Aldrich). SDS‐PAGE used 4%–15% gradient Tris–HCl polyacrylamide precast gels in 1× Tris/Glycine/SDS running buffer (Bio‐Rad). For high molecular weight proteins, overnight wet transfer was performed (20 V, 16 h); for medium and low molecular weight proteins, fast semi‐dry transfer was performed (25 V, 40 min) using a Novex Semi‐Dry Blotter apparatus (Invitrogen). Transfers were onto nitrocellulose membranes (GE Life Sciences). Primary antibodies : mouse monoclonal anti‐pan‐sodium channel (1:500) and anti‐beta‐tubulin (1:10,000; Sigma‐Aldrich); rabbit anti‐total‐lamin (1:2000), anti‐phospho‐lamin (Ser 22, 1:2000), and anti‐alpha‐actinin 2 (1:2000) (Cell Signaling). Secondary antibodies (Thermo Fisher): goat anti‐mouse‐HRP (1:5000) and goat anti‐rabbit‐HRP (1:5000). Primary antibodies were incubated overnight at 4°C. Secondary antibodies were incubated at room temperature for 1 h, developed using SuperSignal West Pico or Femto (Thermo Fisher), and imaged using ChemiDoc XRS+ (Bio‐Rad). Membranes were stripped with Restore Stripping buffer (Thermo Fisher) at 55°C for 15 min followed by shaking at room temperature for 30 min. Antibody and blocking solutions were prepared in 0.1% Tween‐20 in Tris‐buffered saline solution (in mM): NaCl 150, Tris 10 (pH 7.4). Bands were quantified with ImageJ (NIH).

### 2.4 Patch clamp experiments

Cells were harvested using trypsin‐EDTA (Thermo Fisher) for 2 min and transferred to the patch chamber for whole‐cell recording of GFP‐labeled cells, which was performed as previously described (Olaopa et al., 2018; Yu et al., 2014). Whole‐cell configuration was made in bath solution (in mM): NaCl 140, KCl 5, CaCl_2_ 1.8, MgCl_2_ 1, 4‐(2‐hydroxyethyl)‐1‐piperazineethanesulfonic acid (HEPES) 5, and Glucose 10 (pH 7.4 adjusted with NaOH). Pipette resistances were 1.5–3 MΩ; solution (in mM): NaF 10, CsF 110, CsCl 20, ethylene glycol tetraacetic acid (EGTA) 10, and HEPES 10 (pH 7.35 adjusted with CsOH). After achieving a giga‐seal, the test pulse current was nulled by adjusting the pipette capacitance compensator with both fast and slow components. After break‐in, the whole‐cell charging transient was nulled by adjusting whole‐cell capacitance and series resistance. Voltage control protocols were generated with Axopatch 200B amplifier/Digidata 1440A or MultiClamp 700A acquisition system using pCLAMP‐10 software (Molecular Devices). Whole‐cell recording was analyzed using Clampfit 10.2 (Molecular Devices). All experiments were carried out at room temperature. Conductance *G* (V) was calculated by the equation:

$$G\left(V\right)=\frac{I}{\left(V_{m}‐E_{\text{rev}}\right)},$$

where *I* is the peak current, *E*_rev_ is the measured reversal potential, and *V*_m_ is the membrane potential. The normalized peak conductance was plotted as a function of membrane potentials. Steady‐state inactivation was estimated by pre‐pulse protocols (500 ms) from a holding potential of −140 mV. Steady‐state activation and inactivation were fitted with the Boltzmann equation: *y* = [1 + exp ((*V*_m_ − *V*_h_)/*k*)] − 1, where *y* represents variables; *V*_h_, midpoint; *k*, slope factor; and *V*_m_, membrane potential.

### 2.5 Statistical analyses and data availability

The Mann–Whitney–Wilcoxon rank test was performed for patch clamp analyses. One‐way ANOVA test was performed for western blot densitometry. For statistical significance *p* < 0.05 was used. Data are presented as mean ± SE. The data associated with this manuscript will be made available.

## 3 RESULTS

### 3.1 Mutagenesis of Ser 22 residue

We generated *LMNA* phosphorylation plasmids that either mimic loss of phosphorylation in wild type (*S22A‐LMNA*) or constitutive phosphorylation in the *R545H‐LMNA* mutant (*S22D‐R545H‐LMNA*). We selected the *R545H‐LMNA* mutant for our study due to the previously described decrease in Ser 22 phosphorylation. To mimic loss of phosphorylation, we genetically substituted alanine (GCG codon) for serine (TCG codon) residue at position 22 (p.S22A); conversely, to mimic phosphorylation, we genetically substituted aspartic acid (GAC codon) for serine (TCG codon) residue at position 22 (p.S22D). We confirmed successful mutagenesis by sequencing and western blot (Figure 1). Cells that expressed either the *S22A‐LMNA* or *S22D‐R545H‐LMNA* plasmids appeared negative for Ser 22 phosphorylation, indicating the serine residue had been successfully mutated.

### 3.2 Ser 22 Lamin phosphorylation is reduced in *R545H‐LMNA* mutant

**FIGURE 2:**
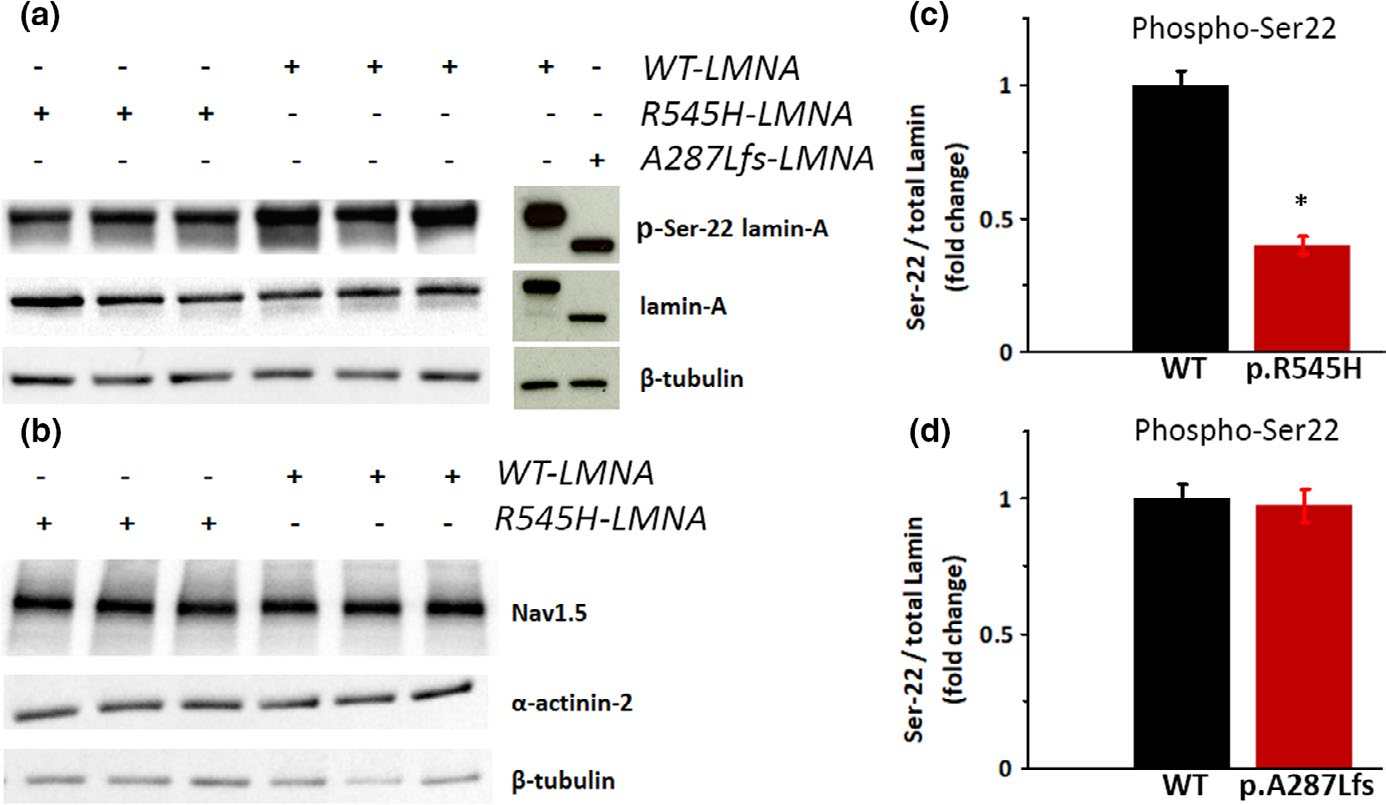
Serine 22 phosphorylation in *LMNA*‐transfected cells. (a, b) Representative western blots of HEK‐293 cells transfected with the combination of plasmids noted. (c, d) Densitometry showing fold change of Ser 22 phosphorylation in: (c) *WT‐LMNA* (*N* = 6) and *R545H‐LMNA* (*N* = 6); *p < 0.05. (d) *WT‐LMNA* (*N* = 6) and *A287Lfs‐LMNA* (*N* = 4); *p* = N.S. Data are mean ± SE

We found that Ser 22 Lamin A/C phosphorylation was reduced by 60% in cells expressing *R545H‐LMNA* compared to wild type (Figure 2a,c). The *A287Lfs‐LMNA* mutation decreased total Lamin A/C levels but did not impact Ser 22 phosphorylation (Figure 2a,d) and was therefore not a subject of this study. *R545H‐LMNA* did not alter Na_v_1.5 whole‐cell protein content (Figure 2b), indicating that the decrease in peak *I*_Na_ was not due to a change in total channel expression. We did not assess localization of Na_v_1.5 to the membrane, and cannot rule out a role for decreased cell surface expression of sodium channels contributing to the decrease in peak *I*_Na_. Alpha‐actinin 2, which has been shown to modulate Na_v_1.5 function via direct interaction (Ziane et al., 2010), was likewise unchanged by *R545H‐LMNA* (Figure 2b).

### 3.3 Ablation of Ser 22 phosphorylation recapitulates *R545H*‐*LMNA* effect on *I*_Na_

**FIGURE 3:**
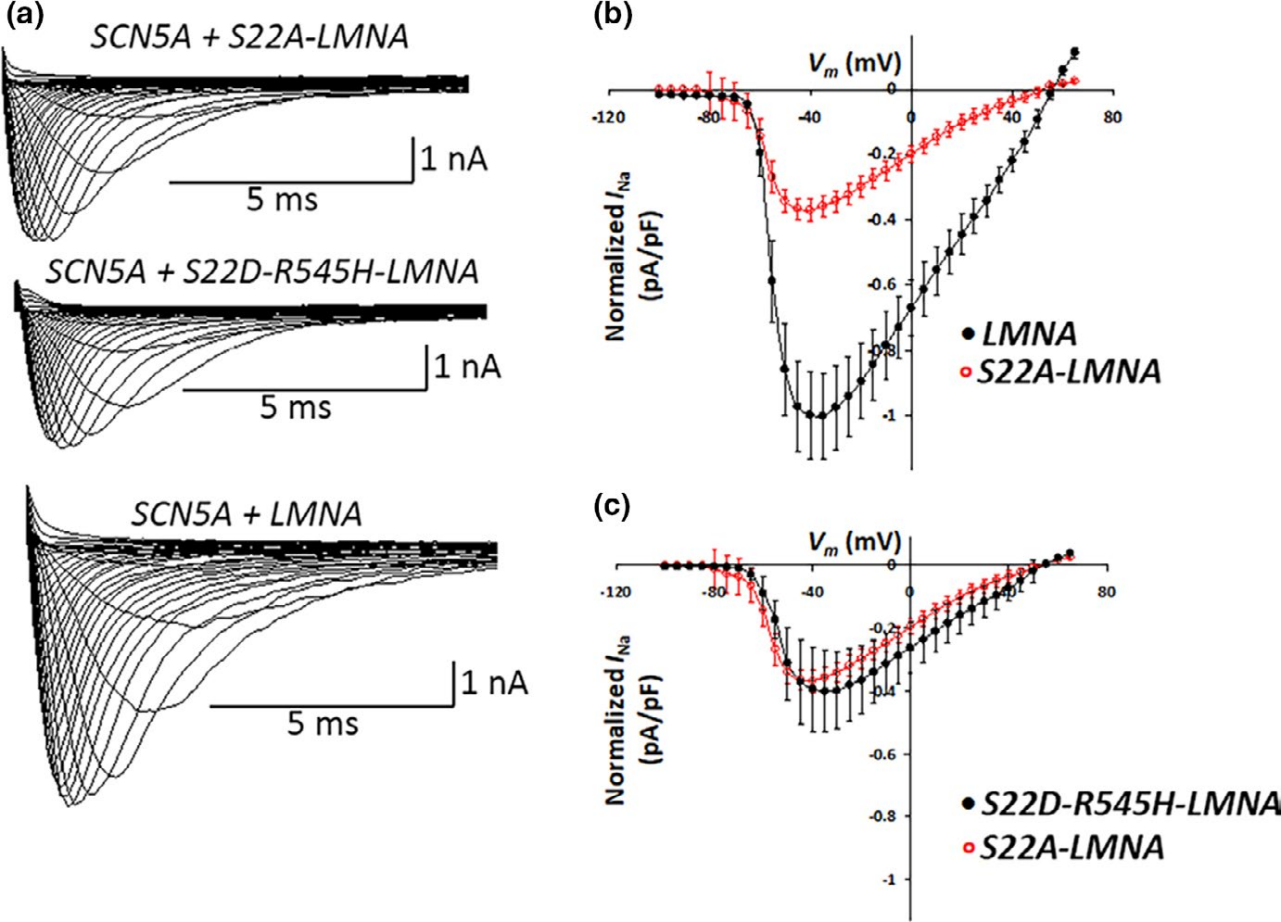
*S22A‐LMNA* phosphorylation mutant alters peak *I*_Na_. (a) Representative superimposed current traces drawn to scale obtained from cells co‐transfected with *SCN5A* and LMNA variants; step‐pulse protocol (inset). (b) I–V relationship comparing cells co‐transfected with *SCN5A* + *WT‐LMNA* (black), *N* = 10 versus *SCN5A* + *S22A‐LMNA* (red), *N* = 10; *p* < 0.0005. Peak current for *SCN5A* + *WT‐LMNA* = 1. (c) I–V relationship comparing cells co‐transfected with *SCN5A* + *S22A‐LMNA* (red), *N* = 10 versus *SCN5A* + *S22D‐R545H‐LMNA* (black), *N* = 7; *p* = N.S. Peak current for *SCN5A* + *WT‐LMNA* is set at 1

**FIGURE 4:**
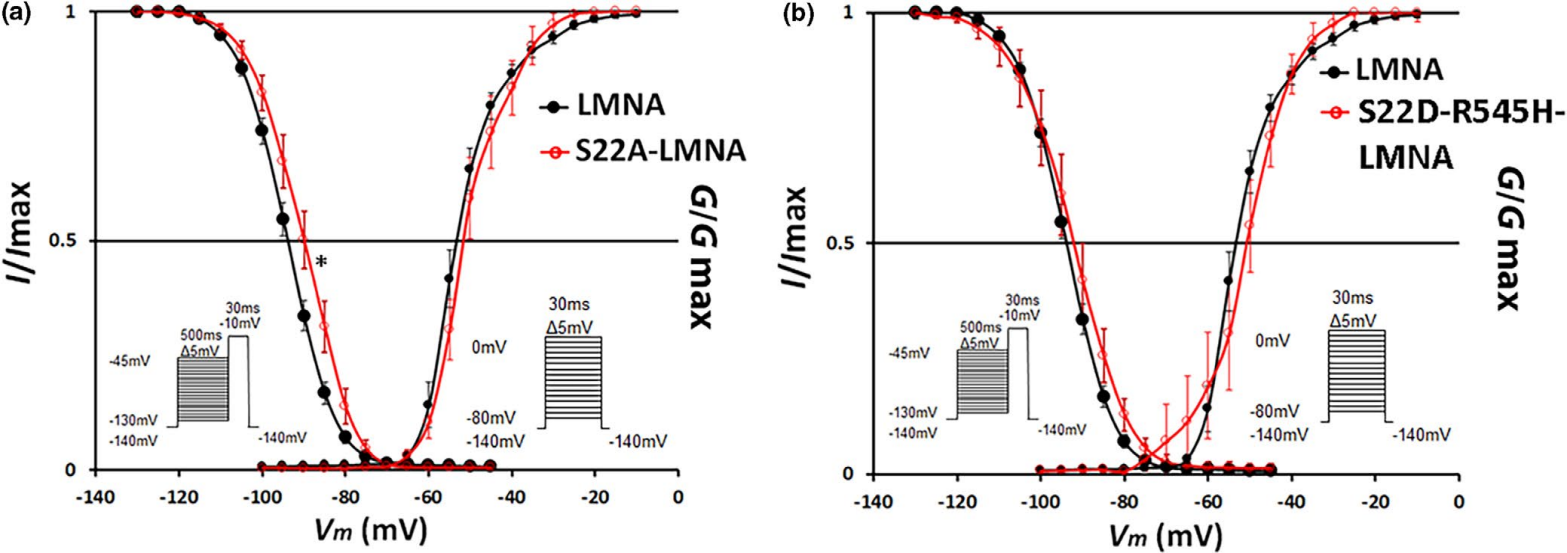
4 *S22A‐LMNA* phosphorylation mutant alters steady‐state inactivation. Voltage‐dependency of the steady‐state inactivation and activation of cells co‐transfected with *SCN5A* + *WT‐LMNA* (black) versus *SCN5A* + *S22A‐LMNA* (red, a) or *S22D‐R545H‐LMNA* (red, b). Inactivation: *SCN5A* + *WT‐LMNA* versus *SCN5A* + *S22A‐LMNA p* < 0.05. No significant differences in other inactivation or activation curves. Insets show pre‐pulse inactivation (left) and step‐pulse activation (right) protocols. Data are mean ± SE

*R545H‐LMNA*, which decreased Ser 22 phosphorylation, also decreased peak *I*_Na_ and shifted voltage‐dependency of steady‐state inactivation rightward, with no effect on steady‐state activation (Olaopa et al., 2018). To determine if Ser 22 Lamin A/C phosphorylation contributed to the regulation of Na_v_1.5, we assessed peak *I*_Na_ in cells transfected with wild‐type *LMNA* or *S22A‐LMNA*, which cannot be phosphorylated on Ser 22. *S22A‐LMNA* significantly decreased peak *I*_Na_ by 60% compared to wild‐type *LMNA* (S22A: −125 ± 12 pA/pF, *N* = 10 vs. wild type: −339 ± 45 pA/pF, *N* = 10, *p* < 0.0005; Figure 3a,b). Similar to the *R545H‐LMNA* mutant (Olaopa et al., 2018), *S22A‐LMNA* also led to a rightward shift of steady‐state of inactivation (*S22A*: *V*_h_ = −88.10 ± 0.51 mV, *N* = 7 vs. wild type: *V*_h_ = −93.20 ± 0.89 mV, *N* = 10, *p* < 0.05) while leaving steady‐state activation unaltered (Figure 4a; Table 1).

**TABLE 1:** Summary of *I*_Na_ parameters

	WT	LBL1 (10 µM)	p.S22A	p.S22D‐R545H
I–V relationship	*N* = 10	*N* = 7	*N* = 10	*N* = 7
Peak *I*_Na_ (pA/pF)	−339 ± 45	−319 ± 26	−125 ± 12***	−137 ± 44***
Steady‐state inactivation	*N* = 10	*N* = 8	*N* = 7	*N* = 6
*V*_h_ (mV)	−93.20 ± 0.89	−95.91 ± 1.72	−88.10 ± 0.51*	−92.60 ± 1.41
*K*	5.62 ± 0.07	7.42 ± 0.17	6.84 ± 0.16	7.23 ± 0.30
Steady‐state activation	*N* = 10	*N* = 7	*N* = 10	*N* = 7
*V*_h_ (mV)	−52.38 ± 0.41	−55.92 ± 1.23	−50.21 ± 1.48	−51.70 ± 1.39
*K*	5.21 ± 0.36	5.37 ± 0.17	5.28 ± 0.22	6.62 ± 0.34

* *p* < 0.05,

*** *p* < 0.0005 (vs. WT).

### 3.4 Mimicking Ser 22 phosphorylation in *R545H‐LMNA* partially restores Na_v_1.5 function

To test if Ser 22 played a role in the mechanism by which *R545H‐LMNA* decreased peak *I*_Na_ (Olaopa et al., 2018), we generated a version of *R545H‐LMNA* that substituted aspartic acid for serine at position 22 (*S22D‐R545H‐LMNA*) to mimic phosphorylation at that site. We then measured peak *I*_Na_ in cells transfected with *S22D‐R545H‐LMNA*. We anticipated that *S22D‐R545H‐LMNA* would restore peak *I*_Na_ to the levels seen with wild‐type *LMNA*. However, a similar loss of peak *I*_Na_ occurred in cells expressing *S22D‐R545H‐LMNA*, indicating that it was unable to rescue the *R545H‐LMNA* effect on peak *I*_Na_ (*S22D‐R545H*: −137 ± 44 pA/pF, *N* = 7 vs. *S22A*: −125 ± 12 pA/pF, *N* = 10, *p* = N.S.; Figure 3a,c). In contrast, *S22D‐R545H‐LMNA* prevented the shift in the steady‐state inactivation of the voltage‐dependency. Cells transfected with *S22D‐R545H‐LMNA* were indistinguishable from wild‐type *LMNA* (Figure 4b; *S22D‐R545H*: *V*_h_ = −92.60 ± 1.41 mV, *N* = 6 vs. wild type: *V*_h_ = −93.20 ± 0.89 mV, *N* = 10, *p* = N.S.). Thus, mimicking Ser 22 phosphorylation partially rescued Na_v_1.5 function disrupted by the *R545H‐LMNA* mutation, as normal voltage‐dependency was restored but peak *I*_Na_ levels were not (Table 1).

### 3.5 LBL1 inhibits Ser 22 phosphorylation but does not affect Na_v_1.5 function

**FIGURE 5:**
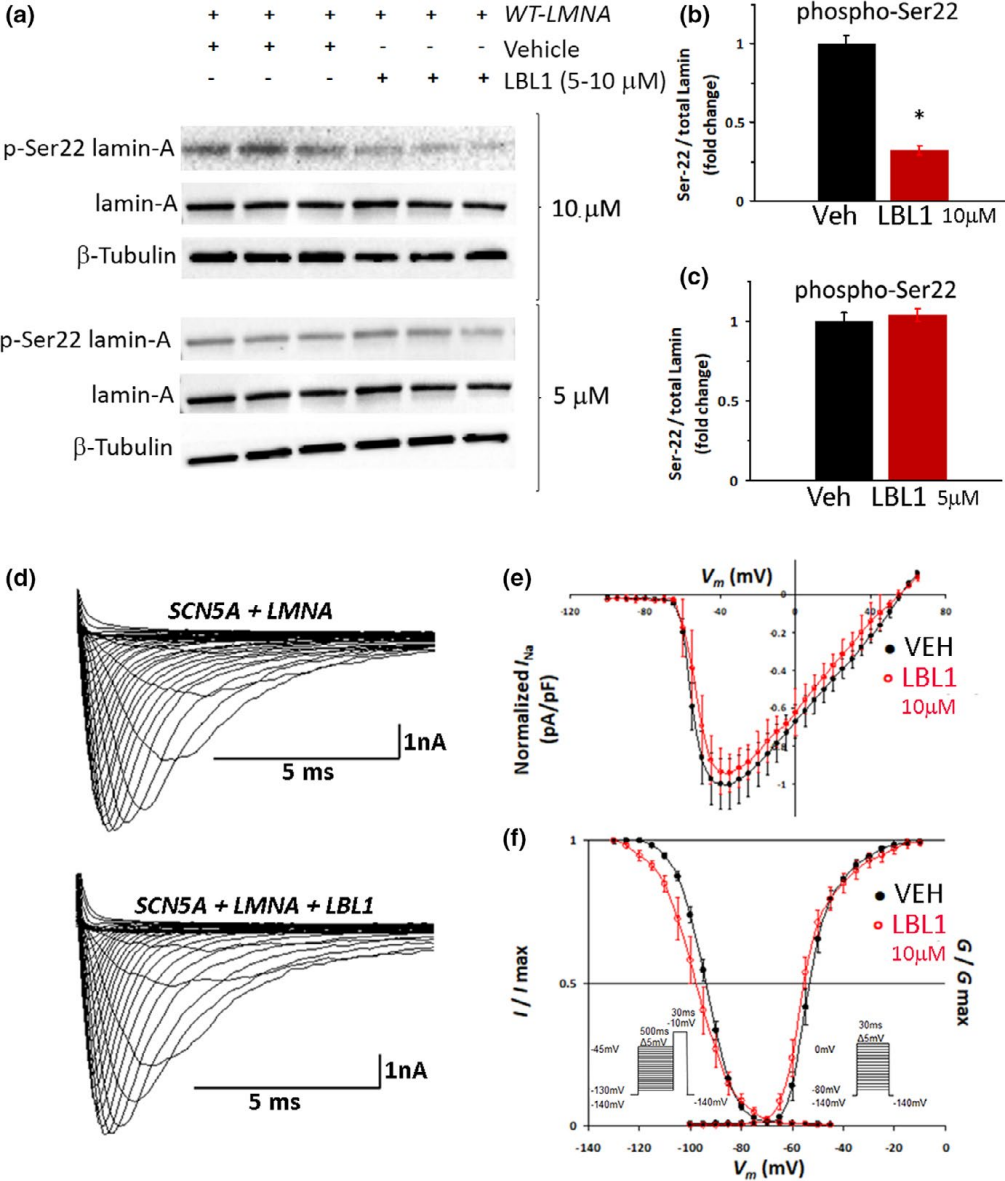
LBL1 decreases Ser 22 Phosphorylation but does not alter *I* *_Na_*. (a) Representative western blot of HEK‐293 cells transfected with *WT‐LMNA* and treated with 10 µM LBL1 (top) or 5 µM LBL1 (bottom). (b, c) Quantification of Ser 22 phosphorylation in cells treated with vehicle, 10 µM (b) or 5 µM (c) LBL1 (red bars). *N* = 6; *p < 0.05. Ser 22 phosphorylation in vehicle‐treated cells was set at 1. (d) Representative superimposed current traces obtained from respective cells. (e) I–V relationship of cells co‐transfected with *SCN5A* + *WT‐LMNA*; vehicle (black), *N* = 10 versus 10 µM LBL1 (red), *N* = 7. Peak current for vehicle is set at 1. (f) Voltage‐dependency of the steady‐state inactivation and activation of cells co‐transfected with *SCN5A* + *WT‐LMNA*. Inactivation: vehicle (black), *N* = 10 versus 10 µM LBL1 (red), *N* = 8; *p* = N.S. Activation: vehicle (black), *N* = 10 versus 10 µM LBL1 (red), *N* = 7; *p* = N.S. Insets show pre‐pulse inactivation (left) and step‐pulse activation (right) protocols. Data are mean ± SE

LBL1, which binds Lamin A/C in the N‐terminal region encompassing Ser 22, was identified based on its ability to inhibit the growth of cancer cells (Chao et al., 2017; Li et al., 2018). LBL1 inhibits the proliferation of cancer cells at micromolar concentrations, so we treated *LMNA*‐transfected HEK‐293 cells with 5 and 10 µM LBL1 to determine if that was sufficient to prevent the phosphorylation of Ser 22. Treating *LMNA*‐transfected cells with 10 µM LBL1 reduced Ser 22 phosphorylation by 65% while 5 µM LBL1 had no effect (Figure 5a–c). To determine the effect of LBL1‐suppressed Lamin A/C phosphorylation on Na_v_1.5 function, we assessed peak *I*_Na_ and voltage‐dependency in cells treated with 10 µM LBL1 compared to vehicle. We anticipated that inhibition of Lamin Ser 22 phosphorylation would lead to changes in Na_v_1.5 function similar to those seen with the *R545H‐LMNA* or *S22A‐LMNA* mutations. However, decreasing Ser 22 phosphorylation by LBL1 in wild‐type Lamin did not change peak *I*_Na_ or steady‐state inactivation or activation (Figure 5d–f; Table 1).

## 4 DISCUSSION

The goal of this study was to understand the effect of Lamin A/C Ser 22 phosphorylation on Na_v_1.5 function, with the aim of identifying a novel therapeutic target for CCD patients with *LMNA* mutations. Our findings indicate that Ser 22 phosphorylation plays a role in Lamin‐mediated Na_v_1.5 modulation and can alter peak *I*_Na_ and voltage‐dependency of the steady‐state inactivation. However, there appears to be a threshold level of phosphorylation necessary in wild‐type Lamin A/C. Partial loss of Ser 22 phosphorylation via the lamin‐binding small molecule LBL1 did not affect Na_v_1.5 function, but complete loss of Ser 22 phosphorylation via genetic substitution had significant effects on channel function that were consistent with the *R545H‐LMNA* variant (Table 1).

Another possible explanation for the discrepancy between the effect of partial and complete loss of Ser 22 phosphorylation is that other Lamin A/C phosphorylation sites, in addition to Ser 22, may play a cumulative role in modulating Na_v_1.5. This alternative interpretation is supported by our findings, which show that mimicking constitutive Ser 22 phosphorylation in the *R545H‐LMNA* mutant only partially restored Nav1.5 function. This suggests that there are other factors and/or phosphorylation sites that also play a role in governing the effect of *R545H‐LMNA* or other *LMNA* mutations on sodium channel function or cell surface expression. To explore this possibility, future studies will be focused on assessing the role of additional Lamin A/C phosphorylation sites and developing chemical and genetic tools that can specifically identify and modulate these sites.

Lamin A/C is an intermediate filament organized into a tripartite structure: a non‐helical N‐terminal head domain encompassing the Ser 22 phosphorylation site; an α‐helical coiled‐coil rod domain encompassing the 287 residue; and an immunoglobulin fold (Ig‐fold) domain at the C‐terminal end encompassing the 545 residue (Ho & Lammerding, 2012; Osmanagic‐Myers et al., 2015). The Ig‐fold domain, which spans residues 430–545, is believed to be involved in Lamin A/C’s protein–protein functional interactions (Dhe‐Paganon et al., 2002; Krimm et al., 2002; Shumaker et al., 2008), including with cytoskeletal linkers that provide the machinery by which sarcolemma proteins like Na_v_1.5 might be modulated (Markandeya et al., 2016; Olaopa et al., 2018; Stroud et al., 2014). Consistent with this, both the *R545H* and *A287Lfs* mutations alter the Ig‐fold domain. *R545H* is a point mutation at the end of the Ig‐fold, a location that has been implicated in other laminopathies including DCM and CCD with loss of peak *I_Na_* (Chan et al., 2016; Liu et al., 2016; Malek et al., 2011; Saj et al., 2010). The *A287Lfs* frame shift mutation alters the sequence from A287 in the rod domain to the premature stop codon in the Ig‐fold. In contrast, LBI1 binds Lamin A/C in the N‐terminal region encompassing Ser 22 (Chao et al., 2017; Li et al., 2018).

Dimerization of Lamin A/C is driven by coiled‐coil formation of its central rod domains (Ho & Lammerding, 2012) (Dhe‐Paganon et al., 2002; Krimm et al., 2002; Stuurman et al., 1998). Lamin A/C dimers assemble head‐to‐tail into polar polymers, which require an overlapping interaction between the head and tail domains (Heitlinger et al., 1992; Sasse et al., 1998). These polymers then laterally assemble in an anti‐parallel fashion into nonpolar filaments (Ben‐Harush et al., 2009). These reports, in addition to our findings, lead us to propose a structural paradigm by which the *R545H* mutation could affect Ser 22 phosphorylation via antiparallel head‐to‐tail interaction with neighboring dimers (graphical abstract). In the case of the *A287Lfs* mutant, which does not result in loss of Ser 22 phosphorylation levels, this head‐to‐tail interaction is likely abolished due to the frame shift and subsequent truncation of this region of the protein. This truncation could explain why modulation of Ser 22 phosphorylation level does not occur in the *A287Lfs* mutant. The complex structure of lamin polymers may also explain why a twofold increase in LBL1 concentration induced a significant loss of Ser 22 phosphorylation. Experimental approaches aimed at exploring these possibilities, while important, are outside the scope and focus of this study.

From a clinical perspective, our results suggest that Lamin A/C phosphorylation may be a potential therapeutic target for patients with specific *LMNA* mutations and CCD. Small molecules that enhance, rather than block, Ser 22 phosphorylation might partially restore Na_v_1.5 function in disease caused by *R545H* and similar *LMNA* mutations. This would mimic the partial rescue of Na_v_1.5 function we observed in cells expressing the *S22D‐R545H‐LMNA* variant. It could also help to improve diagnosis and prognosis in patients with similar *LMNA* mutations by developing tools that could assess their Lamin A/C phosphorylation state.

In summary, our data indicate that Lamin A/C Ser 22 phosphorylation modulates Na_v_1.5 function. This phosphorylation appears to be part of the mechanism by which the *R545H‐LMNA* mutation affects Na_v_1.5 function. To our knowledge, this is the first study to link Lamin A/C phosphorylation and Na_v_1.5 function.

## CONFLICT OF INTEREST

All authors declare no conflict of interest.

## AUTHOR CONTRIBUTIONS

Michael A. Olaopa performed the research. Michael A. Olaopa and Tomohiko Ai performed the analyses. Michael A. Olaopa, Tomohiko Ai, Matteo Vatta, and Beth A. Habecker contributed to conceptualization and design of the study. Bo Chao, Xiangshu Xiao, and Beth A. Habecker contributed analytic tools and resources. Michael A. Olaopa wrote the manuscript with significant contributions from all co‐authors. All authors reviewed and approved the final manuscript.
